# Insights into the role of sterol metabolism in antifungal drug resistance: a mini-review

**DOI:** 10.3389/fmicb.2024.1409085

**Published:** 2024-10-11

**Authors:** Sunita Tanwar, Sapna Kalra, Vinay Kumar Bari

**Affiliations:** Department of Biochemistry, School of Basic Sciences, Central University of Punjab, Bathinda, India

**Keywords:** ergosterol metabolism, oxysterol binding proteins, sterol transfer proteins, sterol regulation, pathogenic fungi

## Abstract

Sterols are essential for eukaryotic cells and are crucial in cellular membranes’ structure, function, fluidity, permeability, adaptability to environmental stressors, and host-pathogen interactions. Fungal sterol, such as ergosterol metabolism, involves several organelles, including the mitochondria, lipid droplets, endoplasmic reticulum, and peroxisomes that can be regulated mainly by feedback mechanisms and transcriptionally. The majority of sterol transport in yeast occurs via non-vesicular transport pathways mediated by lipid transfer proteins, which determine the quantity of sterol present in the cell membrane. Pathogenic fungi *Candida*, *Aspergillus*, and *Cryptococcus* species can cause a range of superficial to potentially fatal systemic and invasive infections that are more common in immunocompromised patients. There is a significant risk of morbidity and mortality from these infections, which are very difficult to cure. Several antifungal drugs with different modes of action have received clinical approval to treat fungal infections. Antifungal drugs targeting the ergosterol biosynthesis pathway are well-known for their antifungal activity; however, an imbalance in the regulation and transport of ergosterol could lead to resistance to antifungal therapy. This study summarizes how fungal sterol metabolism and regulation can modulate sterol-targeting antifungal drug resistance.

## 1 Introduction

Invasive fungal infections increased considerably in immunocompromised or critically ill patients, such as HIV-positive patients, cancer patients undergoing chemotherapy, and organ transplant recipients, which poses a significant global threat to human health (Pfaller and Diekema, 2007; Low and Rotstein, 2011; Garnacho-Montero et al., 2024). Worldwide, 150 million immunocompromised individuals suffer from fungal diseases, which claim the lives of about 1.7 million of them annually (Bongomin et al., 2017; Kainz et al., 2020). Usually, *Candida, Aspergillus*, and *Cryptococcus* spp., infections account for more than 90% of nosocomial fungal infections, primarily affecting immunocompromised individuals (Brown et al., 2012). Numerous species of *Candida* can cause invasive candidiasis, a severe infection that can affect the heart, brain, eyes, bones, blood, and other body parts of patients (Pfaller and Diekema, 2007; McCarty and Pappas, 2016; Lass-Flörl et al., 2024). The prevalence of *Candida albicans* and non-albicans species has grown, particularly in the last two decades (Deorukhkar et al., 2014; Pfaller et al., 2014). This is attributable to a rise in immune-related disorders, the overuse of immunosuppressive medicines, and the prolonged use of medical equipment. The *C. albicans* mainly cause candidiasis, however, several non-albicans species such as *C. parapsilosis, C. glabrata, C. krusei, C. tropicalis, C. dubliniensis, C. lusitaniae* also reported from clinical samples of candidiasis patients (Deorukhkar et al., 2014; Hani et al., 2015; Makanjuola et al., 2018).

**Figure 1:**
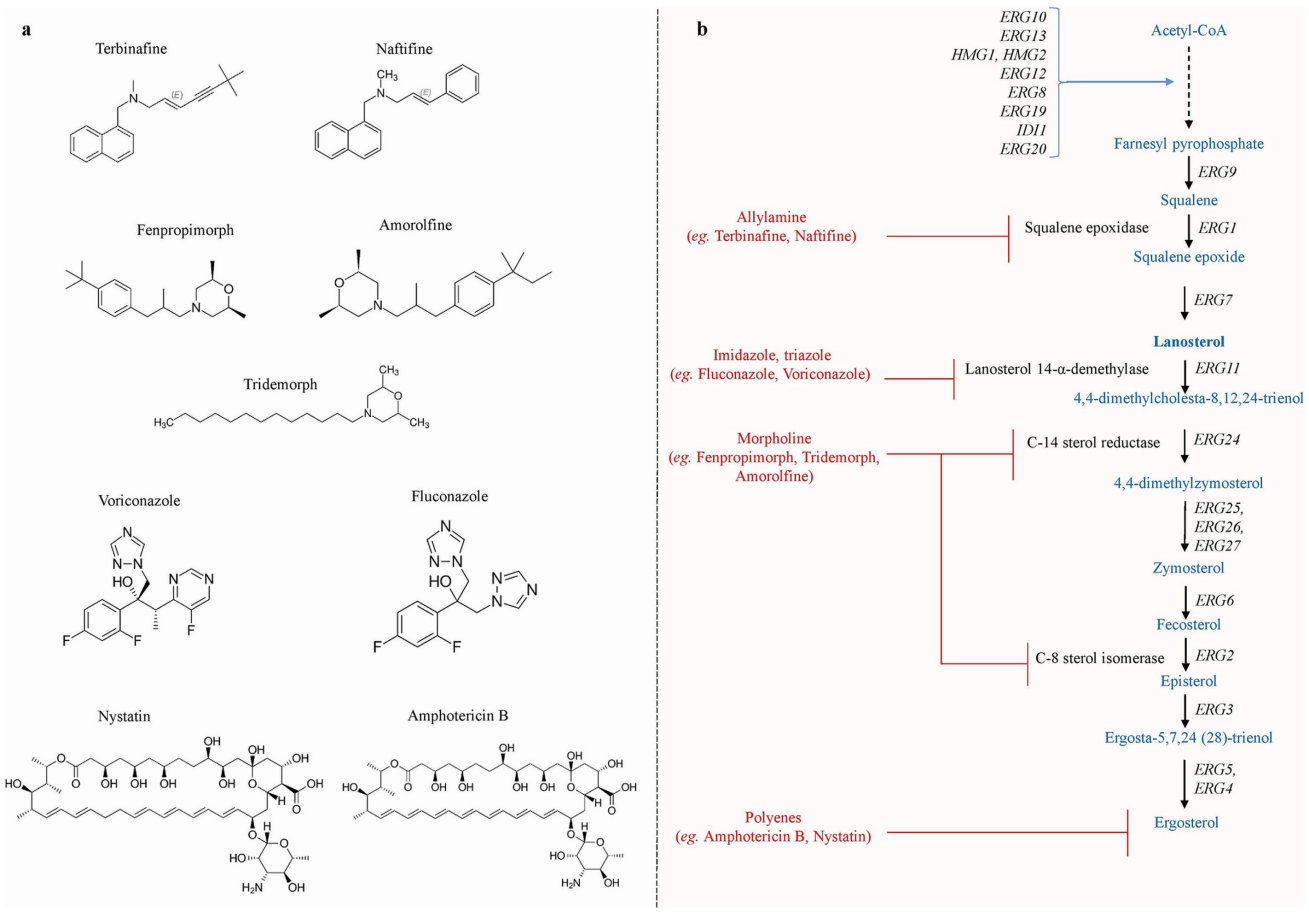
**(a)** The chemical structures of various antifungal drug that specifically targets the ergosterol biosynthesis pathway. **(b)** Schematic representation of ergosterol biosynthesis pathway reported in *S. cerevisiae.* The production of ergosterol molecules begins with the condensation of acetyl-Co and takes place mainly in the ER, whereas farnesyl-PP biosynthesis occurs in the vacuole. Allylamine inhibitors inhibit squalene epoxidase, which is encoded by *ERG1*. Azole inhibitors primarily target *ERG11*, resulting in the generation of hazardous sterols. Morpholines target step catalyzed by *ERG24* and *ERG2*, inhibiting ergosterol production, whereas polyene targets membranes’ ergosterol [Adapted and modified from Onyewu et al., 2003].

Several classes of antifungal drugs with different modes of action have been approved for use in clinical settings to treat fungal infections (Fuentefria et al., 2018). These antifungal drugs include azoles, allylamines, morpholines, polyenes, nucleoside analogs, and echinocandins. Azoles (e.g., fluconazole, voriconazole, and isavuconazole) mainly inhibit the ergosterol biosynthesis pathway enzyme lanosterol-14α-demethylase encoded by *ERG11* and interrupt the ergosterol biosynthesis (Chen and Sobel, 2005; Allen et al., 2015; Lee et al., 2023). Allylamines (e.g., terbinafine and naftifine) inhibit the squalene epoxidase encoded by *ERG1*, and morpholine (e.g., amorolfine, fenpropimorph, and tridemorph) inhibits the biosynthesis of sterol by blocking two successive enzymes (a) C-14 sterol reductase (*ERG24*) and (b) C-8 sterol isomerase (*ERG2*) (Debieu et al., 2000). Polyene (e.g., amphotericin B, nystatin, and natamycin) interacts with ergosterol in the cell membrane, creating pores and causing cell lysis, while echinocandin (e.g., caspofungin and micafungin) mainly inhibits the enzyme *β*-1,3-D-glucan synthase encoded by *FKS1* (Perlin, 2015; Ahmady et al., 2024). Nucleoside analogs, such as 5′-flucytosine (5-FC), are taken up by cytosine permease, a membrane transporter of fungal cells. Cytosine deaminase then transforms the 5-FC into 5-fluorouracil (5-FU), which is its active form. 5-fluorouracil is metabolized to produce 5-fluorouridine monophosphate (5-FUMP) and 5-fluorodeoxyuridine monophosphate (5-FdUMP), which, respectively, blocks RNA and DNA synthesis (Bhattacharya et al., 2020; Sigera and Denning, 2023). The chemical structure of antifungal drugs that selectively target the ergosterol production pathway of *S. cerevisiae* is shown in Figures 1A,B.

The existence of intrinsic, acquired, or clinical resistance poses a significant challenge that limits the potential for the development of novel therapeutics against *Candida* species (Sanguinetti et al., 2015; Chowdhary et al., 2017; Spivak and Hanson, 2018). The rise in clinical isolates of fungal pathogens that are highly virulent and resistant to drugs poses a severe threat to human health (Taei et al., 2019; McDermott, 2022). Public health worldwide is seriously threatened by the emergence of pan-resistant *C. auris* clinical isolates (Lockhart, 2019). Therefore, a fundamental comprehension of the molecular pathways that an appropriate drug can target aids in preventing the development of fungal drug resistance.

Ergosterol, a key sterol in fungal membranes, primarily regulates membrane fluidity, membrane-bound enzyme activity, growth, and other cellular processes, which makes them a potentially valuable target for drug development (Iwaki et al., 2008; Rodrigues, 2018). After being produced in the endoplasmic reticulum (ER), ergosterol is transferred to the plasma membrane (PM), which contains an enormous amount of the cell’s free ergosterol pool (Mesmin et al., 2013). Newly produced ergosterol equilibrates with the PM localized sterol pool, with roughly 10^5^ ergosterol molecules entering and exiting the PM per second (Hu et al., 2017). Sterols are insoluble in water, hence equilibration of free/available sterols between organelles needs lipid transfer proteins between membranes. Soluble lipid transfer proteins take a lipid from a donor membrane and deposit it to an acceptor membrane (Wong et al., 2019). Oxysterol-binding protein (OSBP) and OSBP-related proteins (ORPs) are key candidates of the eukaryotic gene family, including ORP in humans and oxysterol-binding homologous (Osh) proteins in yeast that transfer and regulate sterols and phospholipids between organelle membranes (Weber-Boyvat et al., 2013). Although ergosterol production and transport are well understood in model yeast, little is known about how they contribute to antifungal drugs. This study describes the key steps in ergosterol production and transport pathways that lead to antifungal drug resistance.

## 2 Ergosterol biosynthesis pathway

Ergosterol is mainly synthesized in the ER and transferred to various organelles such as mitochondria, Golgi body, and PM through vesicular and non-vesicular transport mechanisms (Lv et al., 2016; Jordá and Puig, 2020; Zheng Koh and Saheki, 2021). Sterol concentrations are low in the ER but raised in secretory organelles, with the maximum concentration in the PM (Zinser et al., 1993; Hannich et al., 2011). Ergosterol comprises four rings, an acyl side chain, and a hydrophilic hydroxyl group that makes it easy to introduce into membranes. Ergosterol’s structure varies from cholesterol by two additional double bonds (at position C7 and C8) and an extra methyl group (at position C22 and C23) in the side chain (Vanegas et al., 2012).

Sterol biosynthesis in the model yeast *Saccharomyces cerevisiae* begins with the condensing two acetyl-CoA molecules to produce acetoacetyl-CoA, catalyzed by Erg10p (Liu et al., 2019). Erg13p catalyzes the conversion of a third acetyl-CoA to acetoacetyl-CoA, resulting in 3-hydroxy-3-methylglutarylCoA (HMG-CoA) (Miziorko, 2011). HMG-CoA is then reduced to mevalonate by HMG-CoA reductase encoded by Hmg1p and Hmg2p (Basson et al., 1986). Sterol intermediates block these reductases, making this a critical metabolic checkpoint. Erg12p phosphorylates mevalonate during the subsequent process (Oulmouden and Karst, 1991). Erg8p, a phosphomevalonate kinase, further phosphorylates to produce mevalonate-5-pyrophosphate (Tsay and Robinson, 1991). Next, Erg19p, a mevalonate pyrophosphate decarboxylase, decarboxylates isopentenyl pyrophosphate (IPP) (Berges et al., 1997). *IDI1* encodes the IPP isomerase, which converts IPP to dimethylallyl pyrophosphate (DPP). DPP then condenses with another IPP molecule to produce geranyl pyrophosphate, a further addition of IPP results in the formation of farnesyl pyrophosphate (FPP). The geranyl/FPP synthase, Erg20p, catalyzes both processes (Anderson et al., 1989).

Next, squalene synthase (Farnesyl-diphosphate farnesyl transferase) enzyme Erg9 uses two farnesyl-pyrophosphate (farnesyl-PP) molecules to form squalene. The enzymes squalene epoxidase *ERG1* and lanosterol synthase *ERG7* work in tandem to convert squalene to lanosterol. Lanosterol is converted into 4,4-dimethyl-cholesta 8,14, 24-trienol by lanosterol 14α-demethylase encoded by *ERG11* which is further converted into 4,4-dimethyl-zymosterol which is catalyzed by C14 sterol reductase (*ERG24*). 4,4-dimethyl-zymosterol is further converted into zymosterol by involving enzymes such as *ERG25*, *ERG26*, and *ERG27*. Finally, the enzyme C24-methyltransferase (Erg6p) converts the zymosterol into fecosterol. In the next step, fecosterol is converted into episterol, a reaction catalyzed by the C8 isomerase enzyme *ERG2*, and in the last step, episterol is transformed into ergosterol through a complex process involving C5-sterol desaturase (*ERG3*), C22-sterol desaturase (*ERG5*) and C24-sterol reductase (*ERG4*) reactions (Shakoury-Elizeh et al., 2010; Kathiravan et al., 2012; Joshua and Höfken, 2017; Ward et al., 2018; Liu et al., 2019; Vil et al., 2019). The sterol-desaturase enzyme, encoded by *CaERG3*, is known to utilize 14α-methyl-fecosterol in *C. albicans*. This enzyme catalyzes the conversion of 14α-methyl-fecosterol to 14α-methyl-ergosta-8,24(28)-dienol-3,6-diol, a toxic sterol linked to the antifungal activity of triazoles. Ergosterol biosynthesis in yeast depends on oxygen and iron at multiple steps such as enzymatic reactions catalyzed by *ERG1, ERG3, ERG5, ERG11*, and *ERG25* use molecular oxygen and heme as the electron acceptor (Jordá et al., 2022).

## 3 Ergosterol mediated antifungal drug resistance

### 3.1 Allylamine resistance

These days, allylamines such as naftifine and terbinafine are a relatively new class of synthetic antifungal medications against filamentous, dimorphic, yeast-like fungi (Hammoudi Halat et al., 2022). Allylamines act as non-competitive inhibitors mainly by interfering with the initial rate-limiting step of ergosterol biosynthesis catalyzed by squalene epoxidase encoded by *ERG1* (Petranyi et al., 1984). *C. albicans*, and *C. parapsilosis* squalene epoxidase, are susceptible to allylamine, while mammalian liver squalene epoxidase is significantly less sensitive to allylamines (Ryder, 1987; Ryder and Dupont, 1985). Naftifine is a topical fungicidal that is efficient against dermatophytes and is used to treat cutaneous candidiasis. Terbinafine hypersusceptibility was observed in strains overexpressing *ERG1, ERG9*, and *ERG26*. Erg9p converts FPP to produce squalene, a substrate for Erg1p. The overexpression of Erg9p or Erg1p may cause FPP or squalene pools to be diverted toward ergosterol biosynthesis rather than other cellular functions, leading to susceptibility in these strains (Bhattacharya et al., 2018). The *C. albicans erg2Δ/Δ* and *erg24Δ/Δ* mutants are also susceptible to terbinafine, due to the enhanced fluidity and permeability of their PMs (Luna-Tapia et al., 2015). Low terbinafine susceptibility was observed in oropharyngeal *C. albicans* isolates from HIV-positive individuals in a prior investigation (Odds, 2009). Another study also revealed that, except for *C. lusitaniae*, *C. parapsilosis*, and *C. krusei*, other *Candida* spp., isolates were resistant to terbinafine (MIC >32 mg/L) (Tøndervik et al., 2014).

### 3.2 Azole resistance

The azole-antifungal is the most extensive and frequently utilized class of antifungal drugs (Jangir et al., 2023). Azole targets the ergosterol biosynthesis leading to the formation of unusual sterols disrupting the fungal cell membrane. Azoles inhibit the enzyme called lanosterol 14α-demethylase, encoded by *ERG11* (*CYP51*), which is a fungal cytochrome P450-(CYP50) family-dependent enzyme that converts lanosterol into 14α-dimethyl-lanosterol in the ergosterol biosynthesis pathway. The inhibition of this enzyme increases lanosterol and 14α-methyl sterol levels while ergosterol levels decrease. This causes the fungal cell membrane’s typical permeability and fluidity to change, which inhibits the growth of fungal cells (Peyton et al., 2015; Zhang et al., 2019). *ERG2* deletion or *HMG1* overexpression strains showed increased susceptibility when treated with azoles due to lower ergosterol contents (Donald et al., 1997; Bhattacharya et al., 2018). In *S. cerevisiae* and *C. albicans Δerg3* and *Δerg6* deletion strains are resistant to fluconazole due to their inability to manufacture toxic dienol, which accumulates in the wild-type cell after azole treatment (Kodedová and Sychrová, 2015). Fluconazole resistance is also observed in *ERG3* deletion/missense mutant strains of *Cryptococcus neoformans*, *C. glabrata*, *C. parapsilosis*, and *C. albicans* (Branco et al., 2017; Sanglard et al., 2003). Mutation in *ERG25* leads to sterol intermediate accumulation and decreases fluconazole’s binding affinity (Kodedová and Sychrová, 2015; Cavassin et al., 2021). Overexpression of *ERG11* in *C. albicans*, *C. glabrata*, *C. krusei*, and multidrug resistance *C. auris* showed higher resistance to azoles (He et al., 2015; Feng et al., 2017; Bhattacharya et al., 2019).

### 3.3 Morpholine resistance

The ergosterol production pathway enzymes, mainly C14-sterol reductase (*ERG24*), and C8-sterol isomerase (*ERG2*) are targeted by the morpholine class of antifungals e.g., amorolfine, fenpropimorph (FEN) and tridemorph, which leads to the accumulation of abnormal sterols ignosterol (ergosta-8,14 dieno) (Polak, 1988; Lorenz and Parks, 1992; Parks and Casey, 1995). The observation of FEN resistance in strains overexpressing *ERG24* supports that Erg24p is the principal morpholine drug target (Henry et al., 2000). In addition, FEN resistance was observed in disruption mutants of *ERG4*. FEN hypersusceptibility was detected in the strains that overexpressed *NCP1*, *ERG1*, and *HMG1* or in *Δerg3* and *Δerg6* deletion strains (Bhattacharya et al., 2018). It was discovered that *C. albicans* sensitivity to the morpholines can be decreased by overexpressing the *ERG2* or *ERG24* genes (Luna-Tapia et al., 2015). The *erg2Δ* deletion mutant of *C. albicans* was sensitive to FEN, whereas the *erg24Δ* deletion mutant was hypersensitive to amorolfine, FEN, and tridemorph. Mutation in the open reading frame of gene *FEN2* from *S. cerevisiae* that encodes a PM H^+^-pantothenate symporter was shown to be FEN resistance (Stolz and Sauer, 1999). These findings support the theory that the morpholine’s antifungal action depends on the simultaneous inhibition of Erg2p and Erg24p.

### 3.4 Polyene resistance

Polyene class includes amphotericin B (AmB), nystatin, natamycin, and filipins that target PM ergosterol and create a pore in the membrane (Madaan and Bari, 2023). Changes in ergosterol content or the substitution of sterol intermediates are the causes of polyene resistance (Ahmady et al., 2024). Previous research concluded that *C. lusitaniae* may become resistant to AmB due to mutations in or changes in the expression of ergosterol biosynthesis genes (Young et al., 2003). Elevated *ERG6* transcript levels and decreased ergosterol content were observed in *C. lusitaniae* resistant to AmB, indicating mutations or dysregulation in the ergosterol biosynthesis pathway (Bhattacharya et al., 2018). In clinical isolates of *C. glabrata, ERG6*, and *ERG2* are important targets associated with reduced susceptibility to AmB (Ahmad et al., 2019). AmB-resistant isolates also showed lower expression of the *ERG3* gene, which codes for C5 sterol desaturase, suggesting a possible involvement of *ERG3* in the clinical emergence of AmB resistance (Young et al., 2003). *C. neoformans* strains with sterol compositions corresponding to *ERG2* deletion mutant of *S. cerevisiae* are resistant to AmB (Kelly et al., 1994). A previous study in *S. cerevisiae* reported that, as compared to the parental strain, the mutant lacking *ERG4* was slightly more sensitive to nystatin; moreover, the deletion of *ERG2, ERG6,* and, to a lesser extent, *ERG3*, also conferred resistance to this polyene, most likely because of the decreased drug binding affinity for the accumulated fecosterol, zymosterol, and episterol in these mutants (Kodedová and Sychrová, 2015). Natamycin and nystatin-induced loss of inhibition was demonstrated by the loss of double bonds in the B-ring of ergosterol produced by deletions of *ERG3* (5,6 position) and particularly *ERG2* (7,8 position) (te Welscher et al., 2010).

mRNA expression levels of *ERG3* and *ERG6* were decreased but increased for *ERG11* in AmB-resistant isolates of *C. parapsilosis* (Lotfali et al., 2017). The loss of function of *ERG5* (C22 sterol desaturase) or substitution in *ERG11* has been associated with AmB resistance in *C. albicans* (Martel et al., 2010; Vincent et al., 2013). Inactivation of *ERG2* (C8 sterol isomerase) and *ERG6* (C24 sterol methyl-transferase) was reported to have a similar impact on *C. glabrata* (Ahmad et al., 2019). One of the only mechanisms of AmB resistance in *C. neoformans* that has been described involves a mutation that renders *ERG2* inactivated (Kelly et al., 1994). Altered sterol profile due to mutations in several ergosterol biosynthetic pathways genes *ERG11*, *ERG3*, *ERG2*, and *ERG6* also cause AmB resistance in *Candida* species (Vandeputte et al., 2008; Vincent et al., 2013; Carolus et al., 2021; Rybak et al., 2022). Table 1 lists the genes related to ergosterol metabolism and transport pathways that are implicated in resistance to antifungal drugs.

**Table 1:** A list of ergosterol metabolic and transport pathways genes involved in antifungal drug resistance.

Antifungal drugs	Name of the genes involved in ergosterol biosynthesis	Fungal species	Type of genetic approach (Resistance or Sensitive)	References
Amphotericin B	*ERG2, ERG3, ERG5, ERG6, ERG1*	*C. albicans, C. neoformans,C. lusitaniae, S. cerevisiae, C. haemulonii*	Deletion (R)	Ahmad et al. (2019), Lotfali et al. (2017), Martel et al. (2010), and Young et al. (2003)
	*ERG26, ERG6*	*S. cerevisiae*	Overexpression (S)	Bhattacharya et al. (2018)
	*LAM1, LAM2, LAM3*	*S. cerevisiae, C. neoformans*	Deletion (S)	Choy et al. (2003) and Sokolov et al. (2020)
	*LAM2, LAM4*	*S. cerevisiae*	Deletion (S)	
	*ERG2*	*C. albicans*	Double deletion	Luna-Tapia et al. (2015)
	*OSH2*	*S. cerevisiae*	Deletion (R)	Bojsen et al. (2016)
	*OSHB, OSHE*	*A. nidilans*	Deletion(R)	Bühler et al. (2015)
	*OSHC, OSHD*	*A. nidulans*	Deletion(S)	
Azoles	*ERG11*	*C. glabrata, C. albicans, S. cerevisiae*	Overexpression (R)	Bhattacharya et al., 2018, Kodedová and Sychrová (2015), Lotfali et al. (2017), and Young et al. (2003)
	*HMG1, ERG6, ERG3*	*C. lusitaniae, C. albicans, S. cerevisiae*	Deletion (R)	
	*ERG2*	*C. neoformatus*	Deletion(S)	
	*ERG24*	*C. glabrata*	Deletion (R)	
	*HMG1*	*S. cerevisiae*	Overexpression (S)	Bhattacharya et al. (2018)
	*OSH1, ERG3, ERG6, ERG28*	*S. cerevisiae*	Deletion (R)	Anderson et al. (2003)
	*LAM2, LAM4*	*S. cerevisiae*	Deletion (R)	Sokolov et al. (2020)
Fenpropimorph	*ERG2, ERG24*	*C. albicans*	Deletion (S)	Luna-Tapia et al. (2015)
	*HMG1, ERG1*	*S. cerevisiae*	Overexpression (S)	Bhattacharya et al. (2018) and Young et al. (2003)
	*ERG6, ERG3*	*C. lusitaniae, S. cerevisiae*	Deletion (S)	
	*ERG24*	*S. cerevisiae*	Overexpression (R)	
Tridemorph	*ERG2*	*S. cerevisiae*	Deletion (R)	Valachovic et al. (2001)
Terbinafine	*ERG24, ERG2*	*C. albicans*	Deletion (S)	Onyewu et al. (2003) and Luna-Tapia et al. (2015)
	*ERG6*	*C. lusitaniae*	Deletion (S)	Young et al. (2003)
	*ERG9, ERG1, ERG26*	*S. cerevisiae*	Overexpression (S)	Bhattacharya et al. (2018)

## 4 Ergosterol biosynthesis regulation

Ergosterol biosynthesis and degradation must be balanced and regulated to prevent the buildup of free sterols, which can be harmful to cells. Yeast cells have evolved distinct regulatory systems that carefully control the ergosterol composition of lipids. Feedback regulation of ergosterol biosynthesis at the biosynthetic level and transcriptional regulation is responsible for regulating the amount of ergosterol (Jordá and Puig, 2020). Furthermore, the ergosterol pathway enzymes exhibit differential localization. For example, *ERG1* localizes exclusively to the ER to enhance ergosterol synthesis; *ERG6* localizes to the ER, mitochondria, and cytoplasm; however, *ERG1* and *ERG6* also localize in a lipid particle (Leber et al., 1998; Shakoury-Elizeh et al., 2010).

Several enzymes involved in the process of ergosterol biosynthesis work together to control the level of ergosterol produced. Squalene, epoxy squalene, and polyepoxyl squalene, for instance, increase when *ERG27* is inhibited, but not lanosterol, which is identical to that in the *erg7∆* mutant and suggests that *ERG7* and *ERG27* interact genetically (Teske et al., 2008). Subsequent research demonstrated that *ERG27* can interact with *ERG7* and facilitate a relationship between *ERG7* and lipid particles to inhibit *ERG7* degradation; additionally, *ERG27* regulates *ERG7* activity in lipid particles (Layer et al., 2013). Similarly, double deletion mutants of *ERG24* and *ERG4* cannot grow in either nutrient-rich medium YEPD or a synthetic complete medium in the presence of calcium. This phenomenon is also observed when *ERG24* is altered with three additional genes, namely *ERG3*, *ERG5*, and *ERG6* (Luna-Tapia et al., 2015). Furthermore, the expression of ergosterol synthesis is also regulated by the intracellular transportation of ergosterol.

Two endoplasmic reticulum-localized acyl-coenzyme A: sterol acyltransferases, *ARE1* and *ARE2*, which are significantly implicated in sterol esterification, are encoded by *S. cerevisiae* (Yang et al., 1996). Under normal growth conditions, Are2p esterifies the final product, while Are1p primarily esterifies intermediates in sterol biosynthesis (Valachovic et al., 2001). In *S. cerevisiae*, neutral lipids are generated by four enzymes: Are1p and Are2p, which generate stearyl esters; and Lro1p and Dga1p, which generate triacylglycerol and are stored as lipid droplets (Jacquier et al., 2011). *ARE* genes are differently regulated in response to variations in sterol metabolism. The major isoform of the enzyme in a wild-type cell developing aerobically is Are2p. The accumulation of ergosterol pathway intermediates or heme deficiency causes the *ARE1* gene to be up-regulated, while *ARE2* is repressed under heme deficiency. This suggests that the controlled removal of intermediates in the biosynthesis process before they become toxic or contribute to accumulation in the final product is a novel form of sterol homeostasis (Jensen-Pergakes et al., 2001). Despite altered sterol composition, in an ARE1, ARE2 double mutant, stearyl esters (SE) biosynthesis is blocked without any growth defects (Ploier et al., 2015). The double mutant exhibits an increase in free sterols and a decrease in total sterol biosynthesis, suggesting that the formation of SE can also regulate sterol biosynthesis (Ploier et al., 2015). Optimizing culture conditions and metabolic pathway engineering are the two primary techniques for increasing ergosterol productivity since ergosterol biosynthesis is controlled by genes that regulate the biosynthesis and environmental factors (Náhlík et al., 2017). For example, ergosterol biosynthesis can be markedly increased by overexpressing sterol biosynthesis genes (e.g., *ERG1*, *ERG4*, *EGR9*, and *ERG11*) or *ARE2*. Thus, ergosterol biosynthesis regulation is a complicated process influenced by various factors.

**Figure 2:**
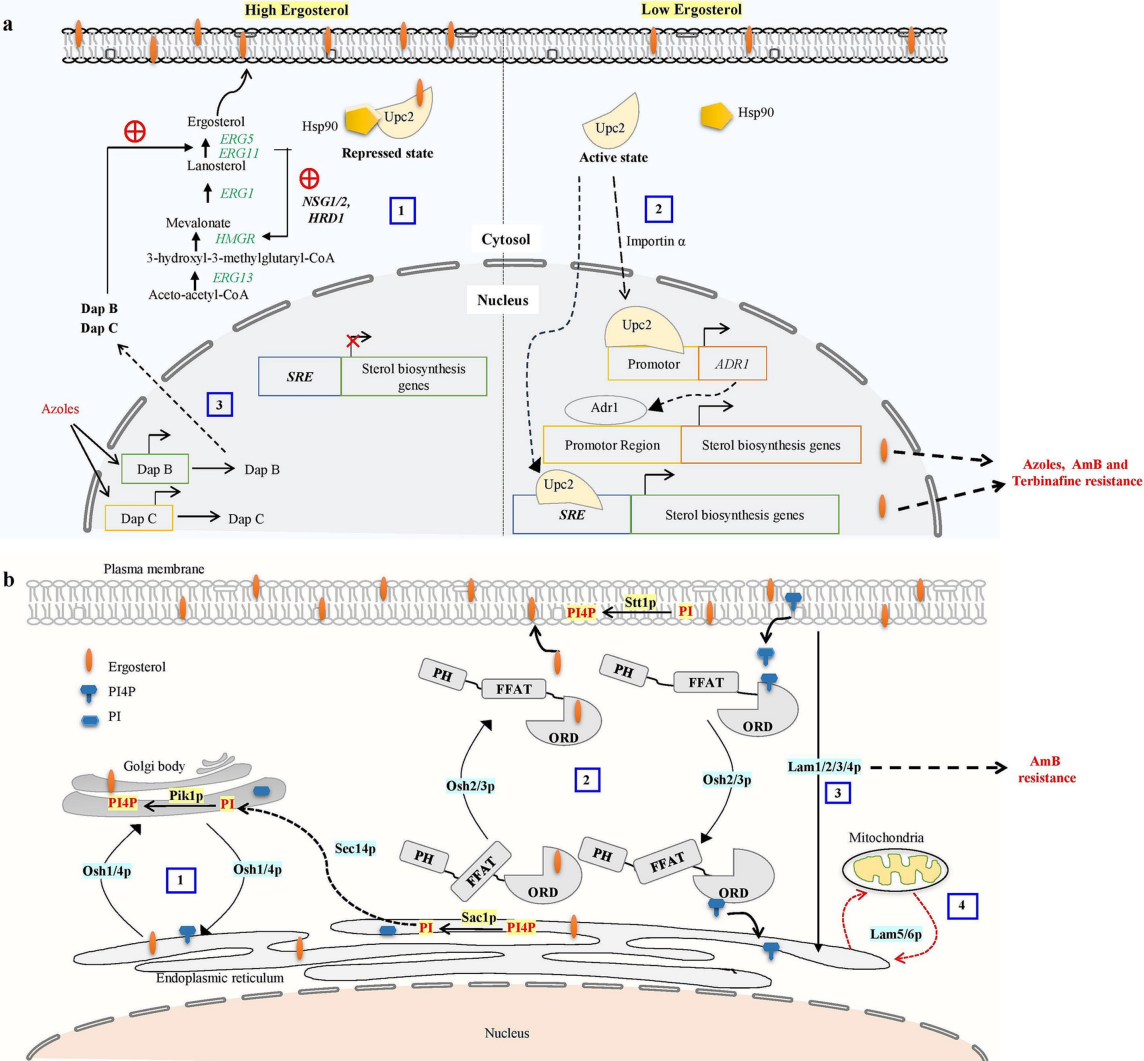
**(a)** Ergosterol biosynthetic pathway regulatory mechanism contributes to antifungal drug resistance. Overabundance of ergosterols may cause HMG-CoA reductase (HMGR) to be degraded via the proteasome, reducing mevalonate synthesis and down-regulating ergosterol biosynthesis. The ER-related degradation (ERAD) pathway mediates the proteasome recognition process of HMGR. The ERAD process primarily initiates HMGR breakdown by *HRD1* and the chaperone proteins *NSG1* and *NSG2* recognizing sterols (Omelchuk et al., 2018). **(1)** In excessive sterol conditions, the ergosterol pathway, specifically transcription factor *UPC2*, binds with ergosterol and Hsp90 and stays in the cytosol as a repressed state. **(2)** Under low ergosterol, dissociation of ergosterol leads to the relocalization of Upc2 from cytosol to the nucleus by nuclear transport proteins such as importins α. transcription factors *UPC2* can bind to the SRE of the sterol biosynthesis genes to promote their expression or Activated Upc2 also triggers the expression of the Adr1 transcription factor, which further serves to direct the expression of ergosterol biosynthesis genes. Activation of Upc2 or Adr1-enhanced azole, AmB, and terbinafine resistance in *Candida* (Shrivastava et al., 2023). **(3)** Three members of the heme-binding damage resistance proteins (Dap) family—*DapA, DapB,* and *DapC* in *A. fumigatus* modulate cytochrome P450 enzymes Erg5 and Erg11 in a coordinated manner and influence azole susceptibility (Song et al., 2016). **(b)** Overview of oxysterol binding protein-mediated sterol transport in *S. cerevisiae.* **(1)** Osh1 and Osh4p acts as a sterol-PI4P exchanger where it acquires the sterol from the donor membrane (ER) and exchange it for a PI4P at the acceptor membrane (trans-Golgi) and then carries the PI4P back to the donor membrane, completing the exchange cycle (Mochizuki et al., 2022). Sec14 protein is involved in the transport of PI from ER to Golgi. PI4P is converted into PI at the ER by Sac1p, and PI is phosphorylated into PI4P at the trans-Golgi by Pik1p (De Saint-Jean et al., 2011). **(2)** *OSH2* and *OSH3* contain the pleckstrin homology (PH) domain in the N-terminal region, the OSBP-related ligand binding domain (ORD) in the C-terminal region, and the (FFAT) motif. These play a part in the counter-transport of ergosterol and PI4P from the ER to the PM and from the PM to the ER, respectively. PI is phosphorylated into PI4P at the PM by Stt1. **(3)** Lam1p, Lam2p, Lam3p, and Lam4p is involved in retrograde transport of sterol. **(4)** *LAM5* and *LAM6* are involved in retrograde transport from ER to mitochondria (Elbaz-Alon et al., 2015; Gatta et al., 2015).

Another important metabolic checkpoint for the biosynthesis of ergosterol is the synthesis of HMG-CoA, which is catalyzed by HMG-CoA reductase (HMGR) (Burg and Espenshade, 2011). Excessive sterols in *S. cerevisiae* can cause HMGR (Hmg2) to be degraded via the ER-related degradation (ERAD) pathway, which lowers mevalonate synthesis and down-regulates sterol production (Espenshade and Hughes, 2007). The ERAD process primarily initiates HMGR degradation with the help of membrane-spanning ubiquitin-protein ligase Hrd1, ubiquitin-conjugating enzyme Ubc7, and the chaperone proteins *NSG1* and *NSG2* (Hampton and Bhakta, 1997; Burg and Espenshade, 2011; Theesfeld and Hampton, 2013; Figure 2A). *ERG1* is also degraded by the ERAD pathway via ubiquitin ligase Doa10 when lanosterol concentration increases to prevent the accumulation of toxic sterol intermediates (Foresti et al., 2013; Huang and Chen, 2023). As a result, ERAD is crucial for preserving cellular sterol homeostasis.

Most fungi including fission yeast *Schizosaccharomyces pombe* and opportunistic pathogen *C. neoformans* contain a homolog of the mammalian sterol regulatory element binding protein (SREBP) known as Sre1, while in *Aspergillus fumigatus* called SrbA (Brown and Goldstein, 1997; Willger et al., 2008; Chang et al., 2009). SREBP-like proteins are activated upon cleavage by SREBP activating protein (SCAP) known as Scp1, that is absent in *A. fumigatus* (Willger et al., 2008). Sre1 localizes to the ER and regulates sterol-specific gene expression (Gómez et al., 2020). Under low sterol conditions, Sre1 gets cleaved and enters the nucleus, binds to sterol regulatory elements (SREs), and increases the expression of sterol-synthesizing genes (Hughes et al., 2005). In addition, hypoxic conditions also induce cleavage of Sre1 and lead to the expression of oxygen-dependent enzymes in the ergosterol biosynthesis pathway including Erg3 and Erg25 (Bien and Espenshade, 2010; Tong et al., 2018). Nevertheless, ergosterol production and absorption are regulated differently depending on the kind of yeast. Fission yeast without Sre1 and Scp1 cannot grow in anaerobic conditions as they cannot manufacture ergosterol at low oxygen levels (Hughes et al., 2005; Stewart et al., 2011; Chong and Espenshade, 2013). Similarly, budding yeast does not take up exogenous sterol, under aerobic or normal growth conditions. But in hypoxic/anaerobic environments, budding yeast does take up exogenous sterol; in fact, sterol absorption is critical to the survivability of budding yeast during anaerobic growth when sterol production is restricted by low oxygen supply (da Costa et al., 2018). Under aerobic circumstances or in the presence of azoles, *C. glabrata* can also absorb cholesterol (Nakayama et al., 2007; Nagi et al., 2013). Moreover, *C. glabrata* can import cholesterol and use it instead of ergosterol when vital genes *ERG1*, *ERG7*, or *ERG11* are knocked down, but this is not the case when *ERG25* and *ERG26* are knocked down (Okamoto et al., 2022).

While budding yeast lacks SREBP homologs, it does have a unique sterol regulatory mechanism that controls ergosterol production. This mechanism involves Upc2 and its paralog Ecm22, both are transcription factors specific to the fungal family able to bind with sterol regulatory elements (SRE) through their amino-terminal Zn_2_Cys_6_ DNA binding domain (Vik and Rine, 2001; Yang et al., 2015). Lethality results from the deletion of Ecm22 and Upc2, indicating that both proteins are crucial for controlling sterol metabolism in budding yeast (Shianna et al., 2001). Upc2 has a hydrophobic pocket in its C-terminal domain that binds to sterol and controls the protein’s transition between the cytosol and nucleus (Vik and Rine, 2001; Marie et al., 2008). Under normal conditions, ergosterol binds with the *UPC2* carboxy-terminal domain causing repression of the *UPC2* transcription factor (Shianna et al., 2001). Under ergosterol depletion or hypoxic conditions, ergosterol ligand dissociation causes conformational changes and the Upc2 transcription factor translocated to the nucleus and activates SRE-containing genes including ergosterol biosynthesis genes, sterol uptake genes (*AVS1*, *PDR11*), and *DAN1/TIR* mannoprotein genes during the anaerobic remodeling of the cell wall (Abramova et al., 2001; Figure 2A).

Dimerization of *UPC2* essential for regulatory function, gain of function mutation in *UPC2* leads to azole resistance while deletion of *UPC2* in *C. albicans* sensitizes them toward azoles (Whaley et al., 2014; Yang et al., 2015). According to previous research, the transcription factors *ECM22* and *UPC2*, the SRE region of the sterol biosynthesis genes enhance their expression under hypoxic conditions (Davies and Rine, 2006; Woods and Höfken, 2016). *AUS1* and *PDR11*, two ATP-binding transporters, can also be expressed in response to *UPC2*, which promotes yeast to absorb sterols from its surroundings. Ergosterol production is further impacted by additional environmental variables such as oxidation, ethanol stimulation, and iron availability (Barchiesi et al., 2005).

## 5 Ergosterol transport pathways

Sterol transport between organelles and release into the medium rely on vesicular or nonvesicular transport pathway (Jacquier and Schneiter, 2012). Under aerobic conditions, yeast does not incorporate exogenous sterols, however, under hypoxia/anaerobic conditions ability to synthesize sterol is decreased which is compensated by the import of sterol from the medium through nonvesicular intracellular trafficking (Lev, 2010). Newly produced lipids are transported non-selectively from the ER to the PM via secretory vesicle flux (Wong et al., 2019). Lipid transfer proteins and sterol binding proteins are two evolutionarily conserved families of proteins that mediate intracellular sterol distribution (Lin et al., 2023). All eukaryotes have the evolutionarily conserved lipid transport proteins (LTPs) known as Oxysterol-binding protein Homology [Osh] in yeast, and Oxysterol-binding Protein [OSBP] and OSBP-Related Protein [ORP] in mammals (Schulz and Prinz, 2007; Lev, 2010; Ngo et al., 2010). The primary biological functions of OSBP and ORP include signaling, vesicular trafficking, lipid metabolism, and non-vesicular transport (Raychaudhuri and Prinz, 2010; Jackson et al., 2016). It has been demonstrated that these proteins can bind and transport different lipids, such as phosphoinositides (PIPs), and sterols (Ngo et al., 2010; Delfosse et al., 2020; Lin et al., 2023; Nakatsu and Kawasaki, 2021).

There is evidence supporting the function of a cytoplasmic nonvesicular protein sterol transporter, and the structure of an oxysterol-binding protein homolog (OSH) in yeast (Osh4p/Kes1p) has been solved, without a ligand and in complexes with many oxysterols, cholesterol, and ergosterol, identifying it as a sterol-binding protein (Schulz and Prinz, 2007). A seven-member oxysterol-binding protein family (*Osh1-7*) in *S. cerevisiae* performs redundant, overlapping roles in sterol metabolism collectively necessary for maintaining intracellular sterol distribution and homeostasis. All seven proteins are demonstrated to have the highest homology within the restricted region of 150–200 amino acid residues that make up OSBP-related domains (ORD) involved in oxysterol binding and intracellular sterol distribution is significantly changed in mutants lacking any of these proteins, which is consistent with an involvement of Osh proteins in intracellular sterol transport (Beh et al., 2001; Beh and Rine, 2004; Schulz and Prinz, 2007).

All these proteins may have a hydrophobic binding tunnel that is important for interaction with sterol. PH domain located at N- the terminal of Osh1p, Osh2p, and Osh3p proteins may control protein targeting to membranes and function as membrane adaptors by interacting with phospholipids (Powis et al., 2023). Osh1p demonstrated a remarkable dual localization at the Golgi and nucleus-vacuole (NV) junction (Levine and Munro, 2001). According to the deletion mapping of Osh1p, the PH domain is shown to be targeting the Golgi while the ankyrin repeat targets the NV junction (Kvam and Goldfarb, 2004). Osh2p is present in the PM, primarily found in the budding region of G1 phase cells around the mother-daughter bud neck of S-phase cells and in the scattered cytoplasmic pool (Levine and Munro, 2001). Osh3p is distributed throughout the cytoplasm and Osh4p is localized into the Golgi membrane (Li et al., 2002). Osh4p binds to phosphatidyl inositol 4-phosphate (PI4P) and its conserved OSBP domain is crucial for Osh4p localization to the Golgi membrane (Kyte and Doolittle, 1982; Rogaski et al., 2010). Osh5 protein is involved in the regulation of ergosterol biosynthesis and facilitates the transfer of phosphatidylserine (PtdSer) to autophagosome membranes (Muramoto et al., 2024), while Osh6p and Osh7p, located in the membrane contact site between the ER and PM, preferentially transporting PtdSer from the ER to PM (Maeda et al., 2013).

Primarily all Osh proteins contain a conserved OSBP-related domain (ORD) made up of an N-terminal lid and a *β*-barrel core, which transports lipids including sterol and phospholipids between membranes. Budding yeast mutants that lack all seven Osh (Osh1-7) proteins are not viable; nevertheless, they can become viable again if they express one of the Osh proteins. Moreover, abrupt Osh protein depletion causes a growth arrest and a massive buildup of sterol in cells (Beh and Rine, 2004). This suggests that Osh proteins perform roles in maintaining cell viability, probably by supporting the distribution of sterol throughout the cell.

Specific Osh proteins mediate the directional transport of sterol between two distinct membrane compartments by exchanging PI4P with sterol. Osh4 and other Osh proteins, including Osh3 and Osh5, mediated sterol transport from the PM to the ER. Osh4 localizes to the Golgi and is involved in controlling the amount of PI4P present in this organelle. Osh4 mutually exclusively binds Sterol and PI4P, and Osh4 counter-transports sterol between artificial membranes *in vitro* in return for PI4P (Rogaski et al., 2010). Previously, it was demonstrated that Osh4p plays a crucial role in maintaining the proper distribution of PI4P in yeast, a function that requires the cooperation of the oxysterol-binding proteins Osh1–Osh7 (LeBlanc and McMaster, 2010; Ling et al., 2014; Figure 2B). Two main PI 4-kinases in budding yeast oversee PI4P production at Golgi and PM. While Stt4 operates at the PM, Pik1 is a lipid kinase in the Golgi apparatus (Audhya et al., 2000). *C. albicans* contains four Osh proteins (Osh2-4 and Osh7), with Osh4 and Osh7 sharing approximately 60% similarity with their *S. cerevisiae* counterparts.

A novel evolutionarily conserved family of LTPs, known as Lam proteins belonging to the steroidogenic acute regulatory protein-related lipid transfer (StART) family was discovered in yeast (Gatta et al., 2015). These StART family proteins contain one or two StART-like domains that are conserved in eukaryotes and involved in transporting sterol between intracellular membranes. Six yeast proteins Ysp1/Lam1, Ysp2/Lam2, Sip3/Lam3, Lam4, Lam5, and Lam6 make up this family in budding yeast exhibit a C-terminal transmembrane region that attaches them to the membrane, and N-terminal StART-like domains, and other pleckstrin-homology (PH) superfamily domain (Gatta et al., 2015; Murley et al., 2015). The budding yeast that lacks Ysp1, Ysp2, or Sip3 exhibits reduced sterol trafficking from the PM to the ER and increased susceptibility to AmB and is rescued by multicopy expression of sterol-binding StART domains (Gatta et al., 2015). This suggests a persistent build-up of PM ergosterol in these yeast mutants. These experiments demonstrate that the Lam proteins help to maintain PM sterol homeostasis in yeast by transporting sterol from the PM to the ER (Figure 2B).

## 6 Conclusion

Finding inhibitors that target the ergosterol pathways in the fungus and can precisely block them without hurting the host is a significant challenge in the research of antifungals. The extensive use of antifungal drugs to treat fungal disease has led to the emergence of multidrug-resistant clinical isolates. Ergosterol alterations in multidrug resistance isolates can be understood by utilizing high throughput approaches, such as metabolomics of clinical isolate, to analyze changes in metabolic pathways and processes that lead to multi-drug resistance. Despite substantial progress in this area, little is known about the relationship between ergosterol transport control and antifungal drug resistance. Enzymes involved in ergosterol biosynthesis, regulation, and transport are necessary for pathogenic fungi to thrive inside their host species. These enzymes also play a crucial role in the virulence of pathogenic fungi. Thus, the pharmaceutical disruption of the ergosterol biosynthesis and transport would impair their ability to respond appropriately to the environmental stress that host cells experience, restricting the proliferation and pathogenicity of pathogenic fungi. Aspergillosis, candidiasis, and cryptococcosis are severe invasive mycoses that have a high mortality rate in immunocompromised patients. Few antifungal drugs are available to treat such invasive infections, and fungus resistance is increasing quickly. Since fungal ergosterol differs structurally from their mammalian counterparts, the ergosterol biosynthesis and transport pathway provides an opportunity to discover novel antifungal drugs. This review improves our understanding of the synthesis, transport, and regulation of ergosterol, which will aid in creating new inhibitors that specifically target ergosterol metabolism.
